# Identifying Unexpected Therapeutic Targets via Chemical-Protein Interactome

**DOI:** 10.1371/journal.pone.0009568

**Published:** 2010-03-08

**Authors:** Lun Yang, Jian Chen, Leming Shi, Michael P. Hudock, Kejian Wang, Lin He

**Affiliations:** 1 Bio-X Center, Key Laboratory for the Genetics of Developmental and Neuropsychiatric Disorders (Ministry of Education), Shanghai Jiao Tong University, Shanghai, China; 2 Institutes of Biomedical Sciences, Fudan University, Shanghai, China; 3 National Center for Toxicological Research, U.S. Food and Drug Administration, Jefferson, Arkansas, United States of America; 4 Institute for Nutritional Sciences, Shanghai Institute of Biological Sciences, Chinese Academy of Sciences, Shanghai, China; University of Queensland, Australia

## Abstract

Drug medications inevitably affect not only their intended protein targets but also other proteins as well. In this study we examined the hypothesis that drugs that share the same therapeutic effect also share a common therapeutic mechanism by targeting not only known drug targets, but also by interacting unexpectedly on the same cryptic targets. By constructing and mining an Alzheimer's disease (AD) drug-oriented chemical-protein interactome (CPI) using a matrix of 10 drug molecules known to treat AD towards 401 human protein pockets, we found that such cryptic targets exist. We recovered from CPI the only validated therapeutic target of AD, acetylcholinesterase (ACHE), and highlighted several other putative targets. For example, we discovered that estrogen receptor (ER) and histone deacetylase (HDAC), which have recently been identified as two new therapeutic targets of AD, might already have been targeted by the marketed AD drugs. We further established that the CPI profile of a drug can reflect its interacting character towards multi-protein sets, and that drugs with the same therapeutic attribute will share a similar interacting profile. These findings indicate that the CPI could represent the landscape of chemical-protein interactions and uncover “behind-the-scenes” aspects of the therapeutic mechanisms of existing drugs, providing testable hypotheses of the key nodes for network pharmacology or brand new drug targets for one-target pharmacology paradigm.

## Introduction

Drug molecules inevitably affect not only their intended protein targets but also other “off-target” proteins as well [1]. These unexpected targets could, in some cases, mediate the physiological effect of a drug, even if the drug is designed specifically to target one particular protein [2]. Several antipsychotics, for example, could trigger similar downstream molecular events when added to the cell culture even without their target, the dopamine receptor, expressed in it [3]. It is generally accepted that chemical-protein interaction is the primary step in triggering molecular events in the biological system when a drug is administered. The identification of unexpected drug-protein interactions could therefore lead to the discovery of new therapeutic targets and therapeutic pathways. There are several strategies in mining such unexpected off-targets, e.g., building new chemical-protein linkages in the known therapeutic target space [2], [4], investigating the pocket shape [5], [6] or sequence identity [7] between the off-target and the known drug target. All these strategies operate on the narrow space of the known drug targets, which represent only a small portion of all human protein space.

Several ‘fishing’ techniques such as BIACORE [8], drug affinity pull-down [9], drug affinity responsive target stability [10] and quantitative proteomics based affinity enrichment [11] can also assess the unexpected drug-protein interactions from a wider protein space. Although not offering a systematic and convincing evaluation of specificity and sensitivity in identifying true or false bindings [12], [13], docking one drug to a multi-protein set has been a logical approach to ‘fishing’ unexpected targets. However, none of the ‘fishing’ techniques described above offer the dramatic progress recently achieved by transcriptomics [3], metabolomics [14] and proteomics [15] in systematically uncovering the molecular events following the administration of a drug into the biological system. One reason might be the inaccuracy of the scoring functions in the ‘fishing’ methodologies. There is no guarantee, for instance, that if the docking score of drug A to protein P1 is lower than A to P2, that P1 has a greater affinity to A than P2 [16]. We therefore hypothesized that investigating the relative strengths of chemical-protein interactions from the ‘-omics’ viewpoint would be much more meaningful than merely comparing the absolute values of a drug's effect on two proteins based on some certain scoring function. Our second hypothesis was that drugs sharing the same therapeutic effect also share the same therapeutic mechanisms by targeting not only on the known target, but also on the same unexpected targets. If the first hypothesis is correct, a more accurate scoring method could be developed that could be applied to the confirmation of the second hypothesis. The two hypotheses require an overview of the drug-protein bindings at the chemical-protein interactomics level.

An interacting model of multi-drug towards multi-protein is therefore introduced in this research, which has successfully been applied in identifying unexpected drug-protein bindings in adverse drug reactions [17]. To test the usefulness of this chemical-protein interactome (CPI) technique on the therapeutic target mining in an effective but low cost way, we chose the DOCK program [18] to construct an *in silico* CPI. We first prepared 10 drug molecules known to treat Alzheimer disease (AD) and 47 drug molecules chosen randomly from Drugbank [19] as the ‘case’ and ‘control’ drugs, hypothesizing that clear differences between the interaction profile of case and control drugs to multi-protein could be observed. The target-mining strategy using this ‘-omics’ data was based on the premise that if the protein was intensively targeted by AD drugs, but did not tend to be targeted by the control drugs it should be prioritized and be measured for its potential therapeutic benefit to AD.

## Results

### Identifying the True Chemical-Protein Interactions Using a Corrected Scoring Method

The docking scores are insufficient to assess absolute chemical-protein interaction strength [16], which might explain why some inverse docking techniques [12], [13] are not widely used in identifying unexpected bindings. To test the performance of our optimization strategies on the drug-protein scorings, selected drug targets from DrugBank [19] and their corresponding structures from the Protein Databank (PDB). Each of the proteins was known to be targeted by at least three FDA-approved drugs with co-crystallized ligands occupying the functional sites. These ligands were also chosen as probe molecules. Pockets without co-crystallized ligands or with heme were excluded, leaving 46 proteins containing 48 pockets for the construction of the test CPI. An *in silico* ‘hybridization’ was performed using DOCK program [18]. Ligands too large to be docked into the pocket of every protein were excluded. In all, an interactome of 44 ligands towards 48 protein pockets were generated in the form of a docking score matrix of 48×44 elements.

**Figure 1:**
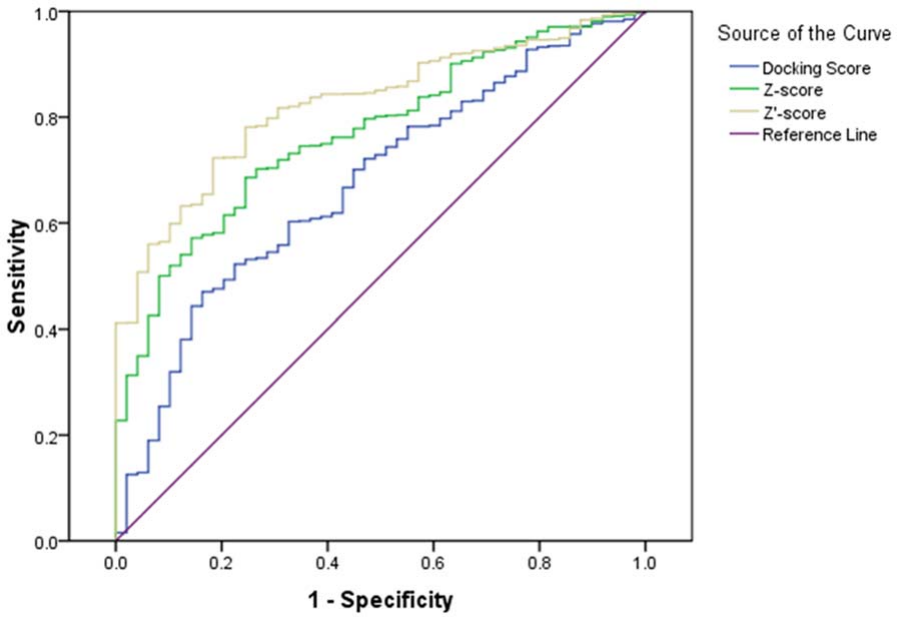
ROC curves representing the power for classifying true and unidentified bindings using docking score, Z-score and Z′-score respectively. The AUC was 0.67, 0.77 and 0.82 for the three scoring systems.

A 2-directional Z-transformation (2DIZ) was then applied to transform the docking score matrix into a Z′-score matrix, where the docking scores were normalized for each drug [20] and then for each protein. Here the original ligand-protein bindings in PDB structures were defined as true bindings, and the others classified as unidentified bindings. The validity of the different scoring systems in separating true and unidentified bindings was expressed in the form of ROC curves (**Fig. 1**). Being close to the reference line, the docking score matrix achieved a poor separating power. However, using the 2DIZ made the AUC reached as high as 0.82. The performance of Z-scores [17] was generally between the two. The predictive accuracy of the Z′-scores may, in fact, be much higher, since some of the unidentified bindings whose Z′-scores were particularly low, might have occurred in any case, and therefore been regarded as false positives. For example, the Z′-score between retinoic acid receptor gamma-2 (1EXX) and retinoic acid (REA) was -3.1, the lowest Z′-score of all drug-protein bindings, was always classified as the true binding while changing the classifying threshold. However, REA was originally embedded in retinoic acid receptor RXR-alpha (1FBY) but not in 1EXX, so the binding of REA to 1EXX was always regarded as a false positive according to the definition of true bindings. Nevertheless, even with this biased evaluation, Z′-scores for 69% of the true bindings compared with only 31% of the unidentified bindings were less than −0.48, which was the threshold when the absolute value of the differential coefficient of the ROC curve reached its minimum, and the sensitivity and specificity are nearly the same. A sensitivity of 0.70 and a specificity of 0.73 is achievable when the threshold is set at −0.48, denoting that a Z′-score less or greater than −0.48 indicates whether or not a binding is likely to be the true binding.

### Linear Model of the Chemical-Protein Interactome Scoring

To give a reasonable explanation to why the Z′-score, not docking score, is more suitable to represent chemical-protein interactions, we put forward a linear model that a docking score X_ij_ can be factorized as:
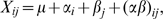
(1)where μ is the mean of docking scores, α_i_ and β_j_ are the assessment of the endogenous contributions of protein factor i and ligand factor j respectively; (αβ)_ij_ is the interactive effect of the two factors, which reflects the true nature of the chemical-protein affinity. Both the ligand and the protein factors are considered as the random effect. Comparing the variances of these effects within the example CPI above, we found that the protein and the ligand effects contributed largely to the variances of the docking scores, and dominated the interactive effect significantly. The mean squares ratios (F values) of the protein and the ligand effect towards the interactive effect are 11.1 (p = 4.2E-67) and 65.8 (p = 0.0) respectively (**File S1**). After applying 2DIZ however, the protein and the ligand factors that contribute to docking scoring are eliminated and the Z′-scores are as follows (see deducing procedures in **File S1**):
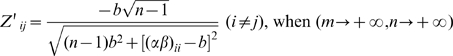


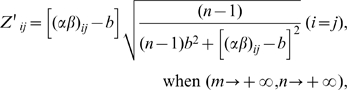
where n and m are the number of ligands and proteins respectively and
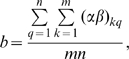
in which denotes that *b* is the mean of all the interactive effects within the matrix. We can see from the above equations that the Z′-score is determined solely by the chemical-protein interactive effect when the elements in the docking score matrix approach positive infinity.

### Constructing the AD Drug-Oriented Chemical-Protein Interactome

**Figure 2:**
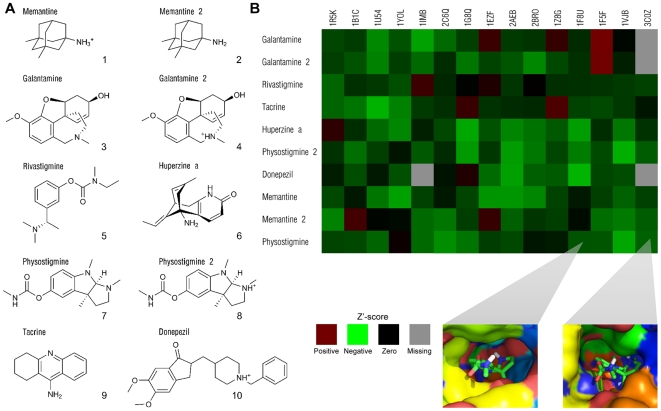
Constructing and mining an AD drug-oriented chemical-protein interactome. (**a**) Structures of 10 case drug molecules. Drug names followed by numbers indicate the derivatives. (**b**) Interactome of these 10 drug molecules towards 15 highlighted proteins in **Table 1**. Proteins are represented by their PDB IDs. Blue, red or white squares represent a Z′-value less than, greater than or equal to zero. Deeper color denotes the greater absolute value. Missing value is presented as a grey box. The accredited drug target of AD (human AChE) is marked with 1F8U. The newly candidate therapeutic target of AD, human HDAC7, is marked with 3C0Z. Binding models of an AD drug, physostigmine, to the pockets of the known (AChE) and unexpected (HDAC7) therapeutic targets of AD are enlarged. Though the shapes and the amino acids sequence of the two pockets are different, the drug accommodates to the two pockets both with steric complementarity and low binding free energy estimated by AutoDock.

Based on the reliability of the Z′-score in specifying true and unidentified bindings, we have initiated an AD drug-oriented CPI that is independent of the test CPI. The chemicals selected here were seven parent AD drugs and three of their major derivatives. To avoid biases in the CPI assessment, we confirmed that the seven drugs did not share significant chemical features (**Fig. 2a**). They were then ‘hybridized’ onto 401 protein pockets (**Table S1**) using DOCK program to generate a case CPI consisting of docking-scores of 401×10 relations. These pockets were derived from third-party databases [19], [21], [22], [23], [24] of protein targets, which were in agreement with the target using pre-defined criteria. To make sure that this target set was not enriched for AD related pathway, we performed KEGG pathway enrichment for all these 401 proteins using DAVID tools [25]. Seven pathways were enriched whose FDR is less than 0.1, but none of them are significantly associated with AD. The control CPI with 401×47 relations was obtained simultaneously through docking all 47 control molecules onto 401 pockets. These 47 drug molecules were randomly chosen from Drugbank, A joint CPI was constructed, and after applying the 2DIZ, the interaction strengths were transformed into a joint Z′-score matrix (401×57 relations). It was then reverted into the AD drug-oriented CPI and the control CPI. For each protein in the CPI, we determined whether they could or could not be targeted by a particular compound if the Z′-score of the interaction was less than or greater than the −0.48. As indicated earlier, Z′-scores beyond this threshold captured 70% of the true bindings and enriched more than three-fold as compared with the unidentified bindings, and the non-parameter hypothesis test we used in the subsequent assessment only required information of this binomial pattern.

### Prioritizing Accredited and Unexpected Therapeutic Targets of AD from the CPI

To identify proteins preferentially interacting with the case drugs, we performed Fisher's exact tests for every protein in comparison to the control. The significance (2-sided) for each of the proteins with relative resk (RR) value (see **Methods**) exceeding one were then calculated and were used as a measure to prioritize the potential drug targets. Proteins with p values less than 0.01 were highlighted (**Fig. 2b** and **Table 1**). Arginase-1 achieved the lowest p value (p = 4.28E-06). This enzyme is involved in the arginine-NO pathway [26], which has just been discovered to be involved in AD pathogenesis [27]. The accredited drug target, acetylcholinesterase (AChE), achieved a p value of 1.3E-3, as 8 of 10 case drugs tended to interact with it whereas only 11 of 47 control drugs bind it. Surprisingly, we discovered that two recently identified therapeutic target families of neurodegenerative disease, the histone deacetylase (HDAC) family and estrogen receptor (ER) family, might have already been involved in the therapeutic pathways of these marketed AD drugs. Both the representative protein of these two families achieved the statistically significant p values (Table 1), indicating that they are preferably targeted by case drugs than control drugs.

**Table 1:** Proteins highlighted from AD drug-oriented CPI using 401 protein set.

PDB ID	Protein Name	*a*	*b*	*c*	*d*	RR	Sig.
2AEB	Arginase-1	10	10	0	37		4.278E-06
1F8U	Acetylcholinesterase	8	11	2	36	8.00	0.001314
1VJB	Estrogen-related receptor gamma	8	11	2	35	7.79	0.001512
1R5K	Estrogen receptor	8	11	2	34	7.58	0.001745
3C0Z	Histone deacetylase 7	6	5	1	22	12.55	0.002037
1F5F	Sex hormone-binding globulin	8	9	2	28	7.06	0.002187
2BRO	Serine/threonine-protein kinase Chk1	7	9	3	38	5.98	0.003079
1IMB	Inositol monophosphatase	7	11	2	36	7.39	0.003179
1YOL	Proto-oncogene tyrosine-protein kinase Src	7	10	3	36	5.35	0.005525
2C6Q	GMP reductase 2	7	11	3	36	5.06	0.007531
1G8Q	CD81 antigen	7	11	3	35	4.93	0.008457
1Z8G	Serine protease hepsin	7	11	3	35	4.93	0.008457
1U54	Activated CDC42 kinase 1	8	14	2	33	6.36	0.009075
1B1C	NADPH–cytochrome P450 reductase	8	14	2	33	6.36	0.009075
1EZF	Squalene synthetase	7	11	3	34	4.80	0.009514

For each protein, *a*, *b*, *c*, *d* values, represents the number of binding (*a* or *b*) and non-binding (*c* or *d*) by case drug molecules or control drug molecules respectively. The two-sided p values were estimated from Fisher's exact test.

**Figure 3:**
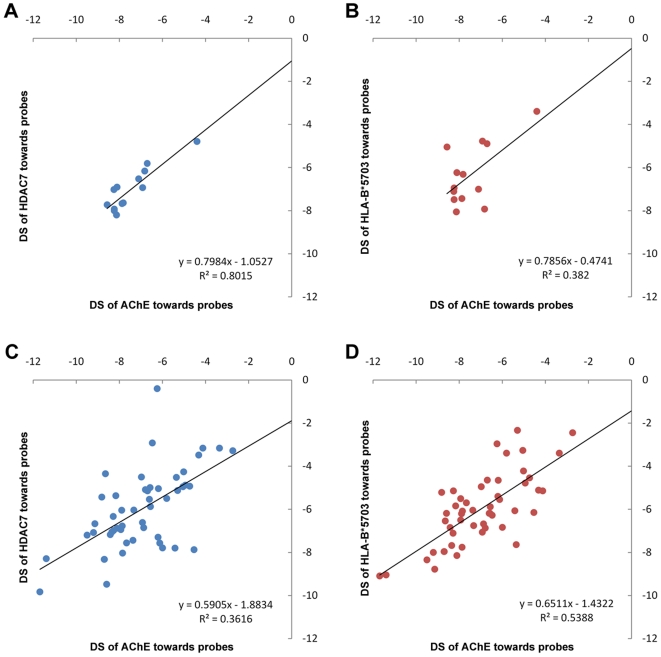
Correlations of docking scores among AChE, HDAC7 and HLA-B*5703 towards probe molecules. The assumed normality and equal variances of docking scores within each group could not be rejectedin statistical tests. The PDB ID of the representative structures of AChE, HDAC7 and HLA-B*5703 were 1F8U, 3Z0Y and 2BVP respectively. See **Table S2** for the detail of their interactomes. (**a, b**) Correlations of docking scores among AChE, HDAC7 and HLA-B*5703 towards 13 probe molecules. (**c, d**) Correlations of docking scores among these three proteins toward 50 control probes.

HDACs, the recently identified therapeutic targets for neurodegenerative diseases [28], [29], [30], [31], catalyze the deacetylation reaction of N^6^-acetyl-lysine of histone. Notably, several biochemical similarities were found between the HDAC and the AChE. For example, the hydrolyzation of acetylcholine catalyzed by AChE is another form of deacetylation. Histidine residue and a Zn^2+^ are involved in the catalytic mechanism of HDAC [32], whereas His447 is also the catalyzing residue in acetylcholine hydrolyzation [33] and the enzyme activity of AChE can be significantly boosted by adding Zn^2+^ cation [34]. These facts indicate similarities in their catalytic mechanisms. Furthermore, A pocket comparison algorithm, SiteSorter, raised a contact similarity score of 0.14 between the active sites of these two enzymes, indicating that there are similarities between the nature of the contacts each site makes with its co-crystallized ligand [35]. To validate the similarity between these two enzymes, we chose another docking program, AutoDock [36], to generate a more comprehensive interactome of two enzymes toward inhibitors and their substrates. Firstly, we chose the substrate of AChE, the acetylcholine, to run a pre-test. AutoDock was able to correctly dock it to the active center of AChE [37] with its acetyl group interacting with two catalyzing residues of AChE (**Fig. S1**) using the docking parameters as described in the **Methods** section. We also confirmed that an inhibitor of HDAC, trichostatin A (TSA) could be docked to the correct position compared with that of the co-crystal ligand (RMSD<0.5). We then chose another HDAC inhibitor (SAHA), together with acetylcholine, TSA and 10 case drug molecules to constitute a ‘probe’ set, which was to ‘hybridize’ onto the two proteins. A control protein, HLA-B*5703, was randomly chosen, hence a CPI of 13×3 relations was constructed. The Pearson correlation coefficient (PCC) between the docking scores of AChE and HDAC7 towards 13 probes was 0.90 (*p* = 3.5e-5, **Fig. 3a**), whereas the PCC between AChE and HLA-B*5703 was 0.62 (*p* = 0.024, **Fig. 3b**). When we randomly chose 50 other molecules from DrugBank (**Table S2**) to constitute a control probe set, the PCC was 0.60 between AChE and HDAC7, and 0.73 between AChE and HLA-B*5703 respectively (**Fig. 3c, d**), indicating that correlation between the interaction profiles was only high given the following two conditions: i) between AChE and HDAC7; ii) using only ligands of AChE or HDAC as the probe. Other members of HDAC family, including human HDAC4, HDAC8 and a yeast HDAC, also showed significant correlation of their interaction profiles with AChE (**Table S3**). However, neither similarity in pocket shape (**Fig. 2b**) nor significant sequence identity in binding site (**Fig. S2**) could be observed between them, implying that the discovery of HDAC could not be made by just comparing the structure or sequence. We can see that the similarity between the pocket of HDAC7 and the AChE is not determined by the pocket shape, but by their interacting pattern with only the probe molecules, namely the AD drugs and the HDAC inhibitors.

The successful recovery of both validated and candidate drug targets of AD, which catalyze similar deacetylating reactions and share a similar interaction profile with probe molecules, is not likely to be achieved by chance. In addition, ERα and ERβ were also highlighted. The ligands of ERα and ERβ are reported to have neuroprotective and anti-inflammatory effects [38], [39] and are promising for AD therapy [40]. They might be the behind-the-scene therapeutic targets of currently marketed AD drugs. Finally, we could thus infer that other proteins highlighted along with these proteins might also be involved in the therapeutic mechanisms of AD, and might serve as the putative therapeutic targets. For example, inositol monophosphatase, which interacts unexpectedly with seven marketed AD drug molecules (p = 3.2E-3), is significantly up-regulated in the AD brain and may be responsible for the pathogenesis of AD [41]. Hence the interactions of AD drug inositol monophosphatase need further investigating.

### The Reliability of the CPI

To test the reliability of the CPI and to better mimic the real situation of the drug space, we chose (from Drugbank) a control set comprising 63 schizophrenia drug molecules (**Table S4**) together with the original 10 AD drug molecules. All of the 73 drug molecules where then ‘hybridized’ onto another published target set to construct a matrix with 73*815 elements. These 63 schizophrenia drug molecules were taken from Drugbank using the same criteria as choosing AD drugs. They were related to one another because all of them could treat the schizophrenia, which is a relevant disease to AD, but the drugs were not known to be related to AD. Separating the AD drugs from these closely related drugs will definitely demonstrate CPI's ability of separating AD drugs from other unrelated drugs. Similar procedures were applied to perform Fisher's exact test for each protein. Proteins with p values less than 0.05 were selected for further investigation (**Table 2**). Three AChEs (1GPK, 1GQS and 2ACE) were included in these 85 highlighted proteins, which showed a significant enrichment from all six AChEs in total 815 proteins (Fisher's exact test p = 0.019). No HDAC protein were highlighted, however, two estrogen receptors (1QKT and 1R5K) and an inosine phosphate (1I9Z) were still being highlighted. In general, even with completely different control set and target set, most of the AD related proteins could still be recalled.

**Table 2:** Proteins highlighted from AD drug-oriented CPI using schizophrenia drugs as the control set.

PDB ID	Protein Name	*a*	*b*	*c*	*d*	RR	Sig.
1Z93	Carbonic anhydrase 3	7	6	3	52	9.87	0.000162
1GPK	Acetylcholinesterase	9	15	1	43	16.50	0.000205
1FKG	Peptidyl-prolyl cis-trans isomerase FKBP1A	8	10	2	43	10.00	0.000356
1J02	Heme oxygenase 1	7	9	2	47	10.72	0.000441
1FVG	Peptide methionine sulfoxide reductase	6	5	3	47	9.09	0.000546
1G8Q	CD81 antigen	7	9	3	51	7.88	0.000763
1WXC	Tyrosinase	4	0	6	41	7.83	0.000840
1EFH	Bile salt sulfotransferase	4	1	6	58	8.53	0.001125
1C9H	Peptidyl-prolyl cis-trans isomerase FKBP1B	6	6	4	53	7.13	0.001144
1R5K	Estrogen receptor	7	11	3	49	6.74	0.001926
1DBK	Ig gamma-1 chain C region secreted form	6	6	4	47	6.38	0.001941
1E4X	TAB2	8	15	2	41	7.48	0.002271
1ME8	Inosine-5′-monophosphate dehydrogenase	7	12	3	50	6.51	0.002405
1GQS	Acetylcholinesterase	8	14	2	38	7.27	0.002511
1NR5	Pentafunctional AROM polypeptide	6	8	4	53	6.11	0.002797
2BFW	GlgA glycogen synthase	7	12	3	48	6.26	0.002900
1C8P	Cytokine receptor common subunit beta	6	8	4	52	6.00	0.003032
2DBL	Ig gamma-1 chain C region secreted form	6	8	4	52	6.00	0.003032
1DBM	Ig gamma-1 chain C region secreted form	6	7	4	47	5.88	0.003078
1DAR	Elongation factor G	7	10	3	40	5.90	0.003489
1JCN	Inosine-5′-monophosphate dehydrogenase 1	7	12	3	46	6.02	0.003515
1FKF	Peptidyl-prolyl cis-trans isomerase FKBP1A	6	8	4	50	5.79	0.003574
1ELA	Chymotrypsin-like elastase family member 1	8	18	2	42	6.77	0.004085
1BZM	Carbonic anhydrase 1	6	7	4	42	5.31	0.004893
1HFW	L-asparaginase	6	7	4	42	5.31	0.004893
1DBB	Ig gamma-1 chain C region secreted form	6	8	4	46	5.36	0.005035
2EU9	Dual specificity protein kinase CLK3	8	18	2	40	6.46	0.005096
1C41	6,7-dimethyl-8-ribityllumazine synthase	6	9	4	49	5.30	0.005591
1YTV	Vasopressin V1a receptor	7	14	3	46	5.44	0.006041
1CPS	Carboxypeptidase A1	6	10	4	52	5.25	0.006115
1H8P_3	Seminal plasma protein PDC-109	4	2	5	40	6.00	0.006322
2BU5	[Pyruvate dehydrogenase [lipoamide]] kinase isozyme 2, mitochondrial	5	5	5	47	5.20	0.006645
2FKY	Kinesin-like protein KIF11	5	5	5	47	5.20	0.006645
1TNJ	Cationic trypsin	5	4	5	40	5.00	0.006978
1ZZD	Ribonucleoside-diphosphate reductase large chain 1	5	5	5	46	5.10	0.007175
5CNA	Concanavalin-A	6	8	3	35	5.43	0.007573
1MCR	IMMUNOGLOBULIN LAMBDA DIMER MCG (LIGHT CHAIN)	6	10	4	48	4.88	0.008387
1GPM	GMP synthase [glutamine-hydrolyzing]	5	7	5	53	4.83	0.010200
2HGS_2	Glutathione synthetase	5	7	5	53	4.83	0.010200
2CMD	Malate dehydrogenase	3	1	7	51	6.21	0.011562
1JNW	Pyridoxine/pyridoxamine 5′-phosphate oxidase	5	7	5	51	4.67	0.011650
1UDH	Uracil-DNA glycosylase	5	7	5	51	4.67	0.011650
2BYU	16.9 kDa class I heat shock protein 2	5	7	5	51	4.67	0.011650
1P8V_1	Platelet glycoprotein Ib alpha chain	6	10	4	44	4.50	0.011693
1I9Z	Inositol-1,4,5-trisphosphate 5-phosphatase 1	6	11	4	47	4.50	0.012141
1GNX	Beta-glucosidase	5	7	5	49	4.50	0.013353
2C6Q	GMP reductase 2	6	12	4	49	4.42	0.013524
1AZM	Carbonic anhydrase 1	5	5	5	38	4.30	0.013847
1FKI	Peptidyl-prolyl cis-trans isomerase FKBP1A	6	12	4	48	4.33	0.014594
2CAB	Carbonic anhydrase 1	4	4	5	45	5.00	0.015191
1BMA	Chymotrypsin-like elastase family member 1	8	23	2	39	5.29	0.015779
1QKT	Estrogen receptor	6	10	4	40	4.13	0.016597
1PTG	1-phosphatidylinositol phosphodiesterase	6	12	4	46	4.17	0.017042
5CPP	Camphor 5-monooxygenase	8	21	2	35	5.10	0.017051
1AHA	Ribosome-inactivating protein momordin I	4	5	5	49	4.80	0.018610
1ILH	Nuclear receptor subfamily 1 group I member 2	6	12	4	44	4.00	0.019975
1NY3	MAP kinase-activated protein kinase 2	6	12	4	44	4.00	0.019975
1JQ9	Phospholipase A2 VRV-PL-VIIIa	6	13	4	47	4.03	0.019992
1PL7	Sorbitol dehydrogenase	6	11	4	40	3.88	0.021754
1TNH	Cationic trypsin	4	5	6	53	4.37	0.021831
1D2F	Protein malY	4	2	5	26	4.13	0.022041
2BO6	Mannosylglycerate synthase	6	13	4	45	3.87	0.023286
7YAS	Hydroxynitrilase	6	11	4	39	3.79	0.023747
1F5F	Sex hormone-binding globulin	7	13	3	34	4.32	0.024106
1A3G	Branched-chain-amino-acid aminotransferase	4	5	6	51	4.22	0.024335
1II5	HYPOTHETICAL PROTEIN SLR1257	3	1	4	24	5.25	0.025306
1HNE	Neutrophil elastase	7	17	3	43	4.47	0.025595
1JS3	Aromatic-L-amino-acid decarboxylase	5	11	4	49	4.14	0.026170
1Q1A	NAD-dependent deacetylase HST2	5	10	5	52	3.80	0.027507
1Q3E	Potassium/sodium hyperpolarization-activated cyclic nucleotide-gated channel 2	5	10	5	52	3.80	0.027507
2ACE	Acetylcholinesterase	7	16	3	38	4.16	0.027537
1CNY	Carbonic anhydrase 2	4	6	6	54	4.00	0.030240
1DGD_1	2,2-dialkylglycine decarboxylase	5	9	5	46	3.64	0.030884
1K74_1	Retinoic acid receptor RXR-alpha	5	10	3	35	4.22	0.032835
2A3Z	Wiskott-Aldrich syndrome protein	4	6	6	52	3.87	0.033525
2PK4	Plasminogen	4	6	6	52	3.87	0.033525
1GL5	Tyrosine-protein kinase Tec	7	20	3	42	3.89	0.033958
1DBJ	Ig gamma-1 chain C region secreted form	5	9	5	44	3.50	0.035372
1YSC	Carboxypeptidase Y	7	17	3	35	3.69	0.036841
1OIT	Cell division protein kinase 2	4	7	6	55	3.70	0.039770
1AVN	Carbonic anhydrase 2	5	10	5	46	3.40	0.040039
1CR1	DNA primase/helicase	5	10	5	46	3.40	0.040039
1HDK_2	Eosinophil lysophospholipase	4	5	5	36	3.64	0.043150
1IJE	Elongation factor 1-alpha	4	7	6	51	3.45	0.048502

PDB IDs marked with a number indicate the pocket number. Refer to the note of Table 1 for the explanation of *a*, *b*, *c*, *d* and Sig.

### CPI Profile of a Drug Reflects Its Therapeutic Effect of AD

One of the concepts of network pharmacology [1] is that drug effect can be mediated by the interactions among drugs towards multiple proteins. Hence drugs sharing the same therapeutic effect would not only share the same drug targets, but might also display a similarity in their interacting profile towards a multi-protein set. If this similarity can be demonstrated from CPI vectors, the efficacy of CPI could be broadened, e.g., the drug efficacy could be predicted by using the docking score vector of a drug towards multi-protein set.

**Figure 4:**
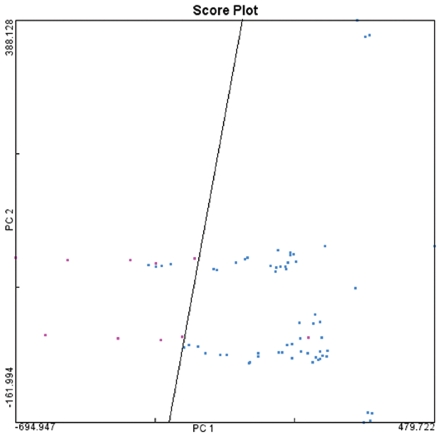
The first two principle components plot of AD and schizophrenia drugs based on their docking score vectors. The figure demonstrates that 10 AD drugs (red) could be basically separated from 63 schizophrenia drugs (blue) linearly.

In the above section, the methodology could highlight the AD related proteins based on the 63 control drugs. Distinguishing AD drugs from the drugs of this AD relevant disease could be a reference of its power in separating drugs of other diseases. Here we applied the principle component analysis (PCA) to explore whether AD drugs could be separated from schizophrenia drugs based on their docking score vectors. The first two components could explain 80.8% of the total variances, and the 10 AD drugs and 63 control drugs could mostly be separated linearly (accuracy = 93.2%, **Fig. 4**). The four ‘false positive’ points from left to right were loxapine, olanzapine, clozapine and molindone respectively. They were not only quite close to the AD drugs in **Fig. 4**, but were also found to be closely linked to AD in their therapeutic effects. For example, olanzapine was effective in treating psychotic and behavioral disturbances in AD [42]; loxapine and molindone had the unlabeled effect of treating psychosis/agitation related to Alzheimer's dementia (http://www.merck.com/mmpe/lexicomp/loxapine.html, http://www.merck.com/mmpe/lexicomp/molindone.html); Clozapine was found for the treatment of agitated-depressed patients with cognitive impairment [43]. The PCA results denoted that the CPI profile of a drug could reflect its therapeutic effect.

## Discussion

It is not sufficient to conduct an accurate assessment of chemical-protein bindings based solely on the original docking scores [16]. From our data we established that the scoring for both the inverse dock [12] and the classical docking method could be improved through systematically mining the CPI. When the Z-transformation was applied for drug j towards multi-protein, the effect of β_j_ was eliminated, leaving only the effect of the α_i_ and (αβ)_ij_; when Z-transformation was applied again for protein i towards multi-drugs on the Z-score, the effect of α_i_ was eliminated, thus only the effect of drug-protein interaction was left. For the inverse docking, the scoring will be inaccurate when α_i_ dominates the (αβ)_ij_. In other word, one cannot be sure that P1 is more affinitive to drug A than P2 if P1 ranks higher than P2 in the docking score list of A towards multi-protein. With virtual screening, on the other hand, it is not certain that D1 is more affinitive to P than D2 when D1 ranks higher than D2 in the scoring list of P's targeting by multi-drug, because β_j_ sometimes dominates (αβ)_ij_. Based on the landscape of the CPI, one can make more reliable judgments for drug-protein interactions. Virtual screening can be considerably improved by the use of the MASC method [20], but the 2DIZ transformation was much more effective than this method on the CPI data (**Fig. 1**). It is anticipated that target screening should consider the difference in the interactome profiles of the library proteins towards multiple drugs; whereas compound screening should investigate the distribution of library molecules towards multiple proteins. Ideally, a CPI comprising of all chemicals and all human macro molecules would be constructed, as deduced in the linear model, the chemical-protein interactive effect would solely be represented by Z′-score if the chemical and target number approached positive infinity.

There may be undiscovered mechanisms which are responsible for the therapeutic effect of the existing AD drugs, and a combined effect on multiple targets may exist. This work demonstrates that the CPI can generate testable hypotheses about the behind-the-scene pharmacology of the existing drugs other than AD drugs. With the help of CPI, candidate key nodes for network pharmacology [1] and new drug targets for one-target pharmacology could be identified. There could be a low cost, high throughput pre-screening step followed by ‘wet’ experiments, and recall of the off-targets would not be hindered by the dissimilarity with the known target in either pocket shape [5], [6] or sequence identity [7]. The identification of unexpected but desired bindings adds to the feasibility of identifying unexpected and unwanted bindings for adverse drug reactions using the CPI methodology [17].

By constructing and mining the CPI, it will be possible not only to harvest unexpected bindings, but also to predict the therapeutic effect or the adverse effect of a drug [17] by uploading the small molecule to a server to construct its CPI signature towards available a human macro molecule set [44]. The CPI signature of the small molecules, whose therapeutic area is unknown, can be compared with the CPI signatures of the existing drugs whose indications are known, providing a potential methodology for pharmaceutical innovation. This is similar to the process of uploading the expression profile of a cell treated by a drug to the connectivity map [3], comparing it with the pre-constructed transcriptomic data of the cell treated with different drugs, and then making a functional linkage between the user's drug and the drugs in the server's database. The expression profile of the cell provides a rich description of cell status; whereas the CPI describes the primary step when a drug is added to the biological system [11], [45]. Knowing which proteins' function is affected by a drug is fundamental, for it could explain the downstream molecular events at the source. The comparison and the combination effect of using two ‘-omics’ platforms in predicting the therapeutic effects and adverse effects of particular drugs will be thoroughly evaluated in follow-up research.

As well as the methodology of CPI introduced above, our research could also inform the search for therapeutic drugs for AD. It is known that several HDAC inhibitors are now in clinical trials aiming at AD therapy, but delivery of the drug to the brain remains a major obstacle [30]. Utilizing the endogenous nature of how the existing AD drugs cross the blood brain barrier might facilitate the design and development of HDAC inhibitors, or even two-target drugs targeting AChE and HDACs to achieve a combined therapeutic effect on AD.

## Materials and Methods

### Preparation of the Protein Pocket Set and the Ligand Set for AD-Oriented CPI

Protein targets were obtained from third-party targetable protein databases [19], [21], [22], [23], [24]. Every pocket had been examined manually when constructing the protein set according to the following criteria: Firstly, the species of the protein should be confined to *Homo Sapiens*; secondly, the pocket must contain the co-crystallized ligand to indicate the targetable state of the protein; thirdly, the pocket should not contain missing residues; fourthly, the protein set should not be redundant. Spheres whose radii ranging from 1.1–1.4Å were generated to fill in the pocket. A grid box was constructed 3–5Å departed from the balls. The case drugs were derived from DrugBank, which were indicated for the treatment of AD in the “Description, Pharmacology, Mechanism_of_Action” fields of the FDA-approved drug table. Control drugs were chosen randomly from DrugBank. The SMILES code of the chemicals was retrieved from PubChem. The minimal energy conformations of chemicals were simulated using CORINA. Charges and hydrogens of proteins and chemicals were added using Chimera [46].

### Construction of the AD-Oriented CPI Using DOCK

The running of the DOCK program and the extraction of the results were controlled by Perl and shell scripts on a Ubuntu™ Linux cluster. The overall docking score of a chemical-protein interaction was calculated using simple energy calculations (electrostatic and van der Waals) with all default parameters used. According to our experience, all the distributions of docking scores in CPI correspond to normal distribution if the data points (docking score >0) are excluded. Docking scores greater than zero were therefore treated as an abnormal value and were excluded. Visualization of CPI scoring matrix was performed using java Treeview [47]. Visualization of chemical-protein interactions were realized using PyMOL.

### The 2-Directional Z-Transformation

Before the 2-directional Z-transformation (2DIZ) was applied to process the original docking-score matrix, a joint CPI (401×57 relations) comprising of a case matrix (401×10 relations) and a control matrix (401×47 relations) was constructed. Here X_ij_ represents the docking-scores of ligand j to protein i in the joint matrix. Firstly, the Z-scores were calculated as:
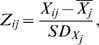
where 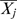 and 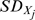 are the mean and the standard deviation of the docking score vector of ligand j. Then the Z-score vector for each protein was normalized with the following formulas, generating the Z′-score matrix.
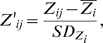
where 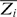 and 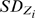 are the mean and the standard deviation of the Z-score vector of protein i.

### Comparing the Variances of between-Subjects Effects within the Test CPI

The type IV method was used to calculate the sum of squares. The normality of the chemical-protein interactive effect was guaranteed by the Kolmogorov-Smirnov test. The F value was computed as MS_c_/MS_cp_ and MS_p_/MS_cp_ respectively, where MS_c_, MS_p_ and MS_cp_ denoted the mean squares of the ligand, protein and the interactive effects.

### Test for Interaction Differences between “Case” and “Control” Drugs for Each Protein

A chemical-protein interaction with Z′-score less or greater than −0.48 was defined as binding or non-binding. For protein i, *a*_i_, *b*_i_, *c*_i_, *d*_i_ values, representing the number of binding (*a*_i_ or *b*_i_) and non-binding (*c*_i_ or *d*_i_) by case or control drug molecules respectively, were counted and the relative risk (*RR*) value was calculated as follows:
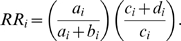
Protein targets with a RR value exceeding one were chosen for Fisher's exact tests, which were carried using an R software package [48].

### Correlation Analysis of CPI Profiles among Protein Targets

The highest absolute value of docking scores generated by AutoDock4 [36] among 50 runs for each chemical-protein interaction was chosen as a reference score. All the default parameters were used when making grids and running AutoDock4, except that the parameter of the genetic algorithm, “Maximum Number of Evals”, was set to 2,000,000. Tests of normality were performed using the Kolmogorov-Smirnov test. Levene's test was applied to the test of equal variances. The Pearson correlation coefficient *r* between protein X and Y was calculated, and the assumption of *r* equals zero was tested using the t-test.

## Supporting Information

Table S1 The 401 human protein pockets set.
(0.41 MB DOC)

Table S2 Interactome of probe molecules towards the tree proteins.
(0.13 MB DOC)

Table S3 Interactome and correlations of docking scores among AChE and other members of HDAC family towards 13 probes.
(0.04 MB DOC)

Table S4 Sixty two schizophrenia drug molecules.
(0.05 MB DOC)

Figure S1 Visualization of the docking result of acetylcholine's interacting with the active center of AChE using AutoDock. The acetyl of acetylcholine interacts with two catalyzing residues (Ser203 and His447), which is accommodate to the catalytic mechanism of AChE.
(1.52 MB TIF)

Figure S2 Comparison of the binding site and sequence identity in active site of AChE and HDAC7. (a, b) Comparison of the docking result of physostigmine to the active site of human AChE and hunan HDAC7. (c, d) Comparison of the docking result of huperzine A to the active site of human AChE and hunan HDAC7. Residues within 6Å of the docked ligand of AChE (PDB ID: 1F8U) are Asp74, Gly82, Thr83, Met85, Trp86, Gly120, Gly121, Gly122, Phe123, Tyr124, Ser125, Gly126, Leu130, Tyr133, Gln202, Ser203, Ala204, Phe295, Phe297, Tyr337, Phe338, Tyr341, Trp439, Pro446, His447, Gly448 and Tyr449. Residues within 6Å of the docked ligand of HDAC7 (PDB ID: 3Z0Y) are His541, Pro542, Glu543, His544, Arg547, Asp626, Pro667, His669, His670, Gly678, Phe679, Asp707, Val708, His709, Phe738, Gly799, Phe800, Asp801, His806, Pro809, Leu810, Gly811, Glu840, Gly841, Gly842 and His843. No significant similarity could be observed within these amino acids between the two proteins.
(0.87 MB TIF)

File S1 Comparing the variances introduced by the ligands and proteins respectively. Deducing procedures of elimination of the protein and the ligand factors.
(0.08 MB DOC)
