# Supplementary material for: Identifying Unexpected Therapeutic Targets via Chemical-Protein Interactome
Source: PLoS One. 2010 Mar 8;5(3):e9568. doi: 10.1371/journal.pone.0009568 (PMC2833192; doi:10.1371/journal.pone.0009568)
Supplement: Table S1 — The 401 human protein pockets set. (0.41 MB DOC) [file pone.0009568.s001.doc]

**Table S1.** The 401 human protein pockets set

| **ID** | **Protein Name** |
| --- | --- |
| **1E51** | Delta-aminolevulinic acid dehydratase |
| **1E96** | Ras-related C3 botulinum toxin substrate 1 |
| **11GS** | Glutathione S-transferase P |
| **1A0L** | Tryptase beta-2 |
| **1A2B** | Transforming protein RhoA |
| **1A3B** | Prothrombin |
| **1A42** | Carbonic anhydrase 2 |
| **1A5Y** | Tyrosine-protein phosphatase non-receptor type 1 |
| **1A7A_1** | Adenosylhomocysteinase |
| **1A7A_2** | Adenosylhomocysteinase |
| **1A7C_1** | Plasminogen activator inhibitor 1 |
| **1A7C_2** | Plasminogen activator inhibitor 1 |
| **1A8M** | Tumor necrosis factor |
| **1A9U** | Mitogen-activated protein kinase 14 |
| **1AD5** | Tyrosine-protein kinase HCK |
| **1ANG** | Angiogenin |
| **1ATK** | Cathepsin K |
| **1AUT** | Vitamin K-dependent protein C |
| **1AXN** | Annexin A3 |
| **1B09** | C-reactive protein |
| **1B1C** | NADPH--cytochrome P450 reductase |
| **1B2T** | Fractalkine |
| **1B56** | Fatty acid-binding protein, epidermal |
| **1B6A** | Methionine aminopeptidase 2 |
| **1BIO** | Complement factor D |
| **1BJ4** | Serine hydroxymethyltransferase, cytosolic |
| **1BKC** | Disintegrin and metalloproteinase domain-containing protein 17 |
| **1BOZ** | Dihydrofolate reductase |
| **1BP1** | Bactericidal permeability-increasing protein |
| **1BQS** | Mucosal addressin cell adhesion molecule 1 |
| **1BX4** | Adenosine kinase |
| **1BYG** | Tyrosine-protein kinase CSK |
| **1BZY** | Hypoxanthine-guanine phosphoribosyltransferase |
| **1C1Y** | Ras-related protein Rap-1A |
| **1C8P** | Cytokine receptor common subunit beta |
| **1C9H** | Peptidyl-prolyl cis-trans isomerase FKBP1B |
| **1CBS** | Cellular retinoic acid-binding protein 2 |
| **1CGH** | Cathepsin G |
| **1CM8** | Mitogen-activated protein kinase 12 |
| **1CSB** | Cathepsin B |
| **1CTR** | Calmodulin |
| **1CVI** | Prostatic acid phosphatase |
| **1CYN** | Peptidyl-prolyl cis-trans isomerase B |
| **1D0A** | TNF receptor-associated factor 2 |
| **1D1T** | Alcohol dehydrogenase class 4 mu/sigma chain |
| **1D2V** | Myeloperoxidase |
| **1D3H_1** | Dihydroorotate dehydrogenase, mitochondrial |
| **1D3H_2** | Dihydroorotate dehydrogenase, mitochondrial |
| **1D4W** | SH2 domain-containing protein 1A |
| **1D5R** | Phosphatidylinositol-3,4,5-trisphosphate 3-phosphatase and dual-specificity protein phosphatase PTEN |
| **1D7K** | Ornithine decarboxylase |
| **1DB1** | Vitamin D3 receptor |
| **1DB4** | Phospholipase A2, membrane associated |
| **1DFV** | Neutrophil gelatinase-associated lipocalin |
| **1DHT** | Estradiol 17-beta-dehydrogenase 1 |
| **1DIA** | Formyltetrahydrofolate synthetase |
| **1DKF** | Retinoic acid receptor alpha |
| **1DMT** | Neprilysin |
| **1DS6** | Ras-related C3 botulinum toxin substrate 2 |
| **1E2D** | Thymidylate kinase |
| **1E3G** | Androgen receptor |
| **1E8Z** | Phosphatidylinositol-4,5-bisphosphate 3-kinase catalytic subunit gamma isoform |
| **1EA6** | Mismatch repair endonuclease PMS2 |
| **1EAX** | Suppressor of tumorigenicity protein 14 |
| **1EEM** | Glutathione S-transferase omega-1 |
| **1EH8** | Methylated-DNA--protein-cysteine methyltransferase |
| **1EK5** | UDP-glucose 4-epimerase |
| **1ELV** | Complement C1s subcomponent |
| **1ERU** | Thioredoxin |
| **1ES7** | Bone morphogenetic protein 2 |
| **1ETA** | Transthyretin |
| **1EXX** | Retinoic acid receptor gamma |
| **1EZF** | Squalene synthetase |
| **1F0R** | Coagulation factor X |
| **1F0Y** | Hydroxyacyl-coenzyme A dehydrogenase, mitochondrial |
| **1F2Q** | High affinity immunoglobulin epsilon receptor subunit alpha |
| **1F3M** | Serine/threonine-protein kinase PAK 1 |
| **1F5F** | Sex hormone-binding globulin |
| **1F5N** | Interferon-induced guanylate-binding protein 1 |
| **1F6O** | DNA-3-methyladenine glycosylase |
| **1F8U** | Acetylcholinesterase |
| **1FBY** | Retinoic acid receptor RXR-alpha |
| **1FE3** | Fatty acid-binding protein, brain |
| **1FGG** | Galactosylgalactosylxylosylprotein 3-beta-glucuronosyltransferase 3 |
| **1FIE** | Coagulation factor XIII A chain |
| **1FO2** | Endoplasmic reticulum mannosyl-oligosaccharide 1,2-alpha-mannosidase |
| **1FPR** | Tyrosine-protein phosphatase non-receptor type 6 |
| **1FQ1** | Cyclin-dependent kinase inhibitor 3 |
| **1FT4** | Tumor necrosis factor receptor superfamily member 1A |
| **1FTA** | Fructose-1,6-bisphosphatase 1 |
| **1FW1** | Maleylacetoacetate isomerase |
| **1FYN** | Proto-oncogene tyrosine-protein kinase Fyn |
| **1G0X** | Leukocyte immunoglobulin-like receptor subfamily B member 1 |
| **1G0Y** | Interleukin-1 receptor type I |
| **1G1T** | E-selectin |
| **1G3M_1** | Estrogen sulfotransferase |
| **1G3M_2** | Estrogen sulfotransferase |
| **1G47** | LIM and senescent cell antigen-like-containing domain protein 1 |
| **1G54** | Carbonic anhydrase 4 |
| **1G55** | tRNA (cytosine-5-)-methyltransferase |
| **1G8I** | Neuronal calcium sensor 1 |
| **1G8Q** | CD81 antigen |
| **1GCZ** | Macrophage migration inhibitory factor |
| **1GFW** | Caspase-3 |
| **1GMN** | Hepatocyte growth factor |
| **1GOS_1** | Amine oxidase [flavin-containing] B |
| **1GOS_2** | Amine oxidase [flavin-containing] B |
| **1GRE** | Glutathione reductase, mitochondrial |
| **1GZP** | T-cell surface glycoprotein CD1b |
| **1GZU** | Nicotinamide mononucleotide adenylyltransferase 1 |
| **1H0C** | Serine--pyruvate aminotransferase |
| **1H1B** | Leukocyte elastase |
| **1H2K** | Hypoxia-inducible factor 1-alpha inhibitor |
| **1H4W** | Trypsin-3 |
| **1H9Z** | Serum albumin |
| **1HA2** | Serum albumin |
| **1HAK** | Annexin A5 |
| **1HDR** | Dihydropteridine reductase |
| **1HE5_1** | Flavin reductase |
| **1HE5_2** | Flavin reductase |
| **1HFC** | Interstitial collagenase |
| **1HKI** | Chitotriosidase-1 |
| **1HMR** | Fatty acid-binding protein, heart |
| **1HNF_1** | T-cell surface antigen CD2 |
| **1HNF_2** | T-cell surface antigen CD2 |
| **1HP7_1** | Alpha-1-antitrypsin |
| **1HP7_2** | Alpha-1-antitrypsin |
| **1HRK** | Ferrochelatase, mitochondrial |
| **1HS6** | Leukotriene A-4 hydrolase |
| **1HSO** | Alcohol dehydrogenase 1A |
| **1HSZ** | Alcohol dehydrogenase 1B |
| **1HT0** | Alcohol dehydrogenase 1C |
| **1HTI** | Triosephosphate isomerase |
| **1HUR** | ADP-ribosylation factor 1 |
| **1HWL** | 3-hydroxy-3-methylglutaryl-coenzyme A reductase |
| **1HYI** | Early endosome antigen 1 |
| **1I0Z** | L-lactate dehydrogenase B chain |
| **1I10** | L-lactate dehydrogenase A chain |
| **1I1N** | Protein-L-isoaspartate(D-aspartate) O-methyltransferase |
| **1I71** | Apolipoprotein(a) |
| **1I7B** | S-adenosylmethionine decarboxylase proenzyme |
| **1I7I** | Peroxisome proliferator-activated receptor gamma |
| **1I92** | Ezrin-radixin-moesin-binding phosphoprotein 50 |
| **1ICE** | Caspase-1 |
| **1IH0** | Troponin C, slow skeletal and cardiac muscles |
| **1IHI_1** | Aldo-keto reductase family 1 member C2 |
| **1IHI_2** | Aldo-keto reductase family 1 member C2 |
| **1IMB** | Inositol monophosphatase |
| **1IRJ** | Protein S100-A9 |
| **1ITU** | Dipeptidase 1 |
| **1IYH** | Glutathione-requiring prostaglandin D synthase |
| **1J1B** | Glycogen synthase kinase-3 beta |
| **1J4I** | Peptidyl-prolyl cis-trans isomerase FKBP1A |
| **1J8F** | NAD-dependent deacetylase sirtuin-2 |
| **1J99** | Bile salt sulfotransferase |
| **1JAP** | Neutrophil collagenase |
| **1JBQ** | Cystathionine beta-synthase |
| **1JCN** | Inosine-5'-monophosphate dehydrogenase 1 |
| **1JD0** | Carbonic anhydrase 12 |
| **1JKL** | Death-associated protein kinase 1 |
| **1JNK** | Mitogen-activated protein kinase 10 |
| **1JUJ_1** | Thymidylate synthase |
| **1JUJ_2** | Thymidylate synthase |
| **1JWH** | Casein kinase II subunit alpha |
| **1K3Y** | Glutathione S-transferase A1 |
| **1K7L** | Peroxisome proliferator-activated receptor alpha |
| **1K86** | Caspase-7 |
| **1KBQ** | NAD(P)H dehydrogenase [quinone] 1 |
| **1KJL** | Galectin-3 |
| **1KPF** | Histidine triad nucleotide-binding protein 1 |
| **1KTA** | Branched-chain-amino-acid aminotransferase, mitochondrial |
| **1L7X_1** | Glycogen phosphorylase, liver form |
| **1L7X_2** | Glycogen phosphorylase, liver form |
| **1LCL** | Eosinophil lysophospholipase |
| **1LN1** | Phosphatidylcholine transfer protein |
| **1LQV** | Endothelial protein C receptor |
| **1LT8** | Betaine--homocysteine S-methyltransferase 1 |
| **1LYW** | Cathepsin D |
| **1M17** | Epidermal growth factor receptor |
| **1M4U** | Bone morphogenetic protein 7 |
| **1MC5_1** | Alcohol dehydrogenase class-3 |
| **1MC5_2** | Alcohol dehydrogenase class-3 |
| **1MLW** | Tryptophan 5-hydroxylase 1 |
| **1MMQ** | Matrilysin |
| **1MQ0** | Cytidine deaminase |
| **1MQB** | Ephrin type-A receptor 2 |
| **1MRQ_1** | Aldo-keto reductase family 1 member C1 |
| **1MRQ_2** | Aldo-keto reductase family 1 member C1 |
| **1MUO** | Serine/threonine-protein kinase 6 |
| **1N7I** | Phenylethanolamine N-methyltransferase |
| **1NAV** | Thyroid hormone receptor alpha |
| **1NFB_1** | Inosine-5'-monophosphate dehydrogenase 2 |
| **1NFB_2** | Inosine-5'-monophosphate dehydrogenase 2 |
| **1NHZ** | Glucocorticoid receptor |
| **1NI4** | Pyruvate dehydrogenase E1 component subunit beta, mitochondrial |
| **1NM8** | Carnitine O-acetyltransferase |
| **1NN6** | Chymase |
| **1NRG** | Pyridoxine-5'-phosphate oxidase |
| **1NRL** | Nuclear receptor subfamily 1 group I member 2 |
| **1NSI** | Nitric oxide synthase, inducible |
| **1NY3** | MAP kinase-activated protein kinase 2 |
| **1O6L** | RAC-beta serine/threonine-protein kinase |
| **1OAT** | Ornithine aminotransferase, mitochondrial |
| **1OF7** | Aldehyde dehydrogenase, mitochondrial |
| **1OGS** | Glucosylceramidase |
| **1OIQ** | Cell division protein kinase 2 |
| **1OIZ** | Alpha-tocopherol transfer protein |
| **1OLM** | SEC14-like protein 2 |
| **1OLS** | 2-oxoisovalerate dehydrogenase subunit alpha, mitochondrial |
| **1ONQ** | T-cell surface glycoprotein CD1a |
| **1OPL** | Proto-oncogene tyrosine-protein kinase ABL1 |
| **1ORE** | Adenine phosphoribosyltransferase |
| **1OSH** | Bile acid receptor |
| **1OTH** | Ornithine carbamoyltransferase, mitochondrial |
| **1P49** | Steryl-sulfatase |
| **1P4M_1** | Riboflavin kinase |
| **1P4M_2** | Riboflavin kinase |
| **1P5J** | L-serine dehydratase |
| **1PIC** | Phosphatidylinositol 3-kinase regulatory subunit alpha |
| **1PIN** | Peptidyl-prolyl cis-trans isomerase NIMA-interacting 1 |
| **1PL7** | Sorbitol dehydrogenase |
| **1POZ** | CD44 antigen |
| **1PQ2** | Cytochrome P450 2C8 |
| **1PSO** | Pepsin A |
| **1PT9** | NAD(P) transhydrogenase, mitochondrial |
| **1PTW** | cAMP-specific 3',5'-cyclic phosphodiesterase 4D |
| **1PUB** | Ganglioside GM2 activator |
| **1Q11** | Tyrosyl-tRNA synthetase, cytoplasmic |
| **1Q1Z** | Sulfotransferase family cytosolic 2B member 1 |
| **1Q4O** | Serine/threonine-protein kinase PLK1 |
| **1QAB** | Retinol-binding protein 4 |
| **1QCY** | Integrin alpha-1 |
| **1QIA** | Stromelysin-1 |
| **1QIP** | Lactoylglutathione lyase |
| **1QJA** | 14-3-3 protein zeta/delta |
| **1QMV** | Peroxiredoxin-2 |
| **1QPC** | Proto-oncogene tyrosine-protein kinase LCK |
| **1QR6** | NAD-dependent malic enzyme, mitochondrial |
| **1R47** | Alpha-galactosidase A |
| **1R4L** | Angiotensin-converting enzyme 2 |
| **1R55** | ADAM 33 |
| **1R5K** | Estrogen receptor |
| **1R6T** | Tryptophanyl-tRNA synthetase, cytoplasmic |
| **1R74** | Glycine N-methyltransferase |
| **1R82** | Glycoprotein-fucosylgalactoside alpha-galactosyltransferase |
| **1R9O** | Cytochrome P450 2C9 |
| **1RD4** | Integrin alpha-L |
| **1RFN** | Coagulation factor IX |
| **1RT9** | Purine nucleoside phosphorylase |
| **1S8C** | Heme oxygenase 1 |
| **1SD2** | S-methyl-5'-thioadenosine phosphorylase |
| **1SG0_1** | Ribosyldihydronicotinamide dehydrogenase [quinone] |
| **1SG0_2** | Ribosyldihydronicotinamide dehydrogenase [quinone] |
| **1SMO** | Triggering receptor expressed on myeloid cells 1 |
| **1SQN** | Progesterone receptor |
| **1SWX** | Glycolipid transfer protein |
| **1T40_1** | Aldose reductase |
| **1T40_2** | Aldose reductase |
| **1TDI** | Glutathione S-transferase A3 |
| **1TG2** | Phenylalanine-4-hydroxylase |
| **1TVO** | Mitogen-activated protein kinase 1 |
| **1TYL** | Insulin |
| **1U54** | Activated CDC42 kinase 1 |
| **1UDT** | cGMP-specific 3',5'-cyclic phosphodiesterase |
| **1UHL_1** | Retinoic acid receptor RXR-beta |
| **1UHL_2** | Retinoic acid receptor RXR-beta |
| **1UKI** | Mitogen-activated protein kinase 8 |
| **1UMK** | NADH-cytochrome b5 reductase 3 |
| **1UOU** | Thymidine phosphorylase |
| **1UPV** | Oxysterols receptor LXR-beta |
| **1UWJ** | B-Raf proto-oncogene serine/threonine-protein kinase |
| **1UZF** | Angiotensin-converting enzyme |
| **1V04** | Serum paraoxonase/arylesterase 1 |
| **1VCU** | Sialidase-2 |
| **1VJ5** | Epoxide hydrolase 2 |
| **1VJA** | Urokinase-type plasminogen activator |
| **1VJB** | Estrogen-related receptor gamma |
| **1W0H** | Histone mRNA 3'-exonuclease 1 |
| **1W22** | Histone deacetylase 8 |
| **1W7N** | Kynurenine--oxoglutarate transaminase 1 |
| **1WDA** | Protein-arginine deiminase type-4 |
| **1WOK** | Poly [ADP-ribose] polymerase 1 |
| **1WWA** | High affinity nerve growth factor receptor |
| **1WWB** | BDNF/NT-3 growth factors receptor |
| **1WWC** | NT-3 growth factor receptor |
| **1X0O** | Aryl hydrocarbon receptor nuclear translocator |
| **1X50** | Galectin-4 |
| **1XAP** | Retinoic acid receptor beta |
| **1XBA** | Tyrosine-protein kinase SYK |
| **1XF0_1** | Aldo-keto reductase family 1 member C3 |
| **1XF0_2** | Aldo-keto reductase family 1 member C3 |
| **1XLV** | Cholinesterase |
| **1XMI** | Cystic fibrosis transmembrane conductance regulator |
| **1XOS** | cAMP-specific 3',5'-cyclic phosphodiesterase 4B |
| **1XQZ** | Proto-oncogene serine/threonine-protein kinase Pim-1 |
| **1XU9_1** | Corticosteroid 11-beta-dehydrogenase isozyme 1 |
| **1XU9_2** | Corticosteroid 11-beta-dehydrogenase isozyme 1 |
| **1XVP** | Nuclear receptor subfamily 1 group I member 3 |
| **1XWK** | Glutathione S-transferase Mu 1 |
| **1Y0S** | Peroxisome proliferator-activated receptor delta |
| **1Y0X** | Thyroid hormone receptor beta |
| **1YB5** | Quinone oxidoreductase |
| **1YET** | Heat shock protein HSP 90-alpha |
| **1YOL** | Proto-oncogene tyrosine-protein kinase Src |
| **1YOW** | Steroidogenic factor 1 |
| **1YV5** | Farnesyl pyrophosphate synthetase |
| **1YVJ** | Tyrosine-protein kinase JAK3 |
| **1Z57** | Dual specificity protein kinase CLK1 |
| **1Z6J** | Coagulation factor VII |
| **1Z6T** | Apoptotic protease-activating factor 1 |
| **1Z8G** | Serine protease hepsin |
| **1Z93** | Carbonic anhydrase 3 |
| **1ZBQ** | 3-alpha,7-alpha,12-alpha-trihydroxy-5-beta-cholest-24-enoyl-CoA hydratase |
| **1ZJK** | Mannan-binding lectin serine protease 2 |
| **1ZKK_1** | Histone-lysine N-methyltransferase SETD8 |
| **1ZKK_2** | Histone-lysine N-methyltransferase SETD8 |
| **1ZSX** | Voltage-gated potassium channel subunit beta-2 |
| **1ZT3** | Insulin-like growth factor-binding protein 1 |
| **1ZV4** | Regulator of G-protein signaling 17 |
| **1ZXM** | DNA topoisomerase 2-alpha |
| **1ZXQ** | Intercellular adhesion molecule 2 |
| **2A3I** | Mineralocorticoid receptor |
| **2AB6** | Glutathione S-transferase Mu 2 |
| **2AC3** | MAP kinase-interacting serine/threonine-protein kinase 2 |
| **2AEB** | Arginase-1 |
| **2AHE** | Chloride intracellular channel protein 4 |
| **2ANY** | Plasma kallikrein |
| **2AOU** | Histamine N-methyltransferase |
| **2AUH** | Insulin receptor |
| **2AVD** | Catechol-O-methyltransferase domain-containing protein 1 |
| **2AYO** | Ubiquitin carboxyl-terminal hydrolase 14 |
| **2B3K** | Methionine aminopeptidase 1 |
| **2B4Y_1** | NAD-dependent deacetylase sirtuin-5 |
| **2B4Y_2** | NAD-dependent deacetylase sirtuin-5 |
| **2B7A** | Tyrosine-protein kinase JAK2 |
| **2BH9** | Glucose-6-phosphate 1-dehydrogenase |
| **2BIY** | 3-phosphoinositide-dependent protein kinase 1 |
| **2BRO** | Serine/threonine-protein kinase Chk1 |
| **2BU5** | [Pyruvate dehydrogenase [lipoamide]] kinase isozyme 2, mitochondrial |
| **2BX8_1** | Serum albumin |
| **2BX8_2** | Serum albumin |
| **2BX8_3** | Serum albumin |
| **2BXF** | Serum albumin |
| **2C2Z** | Caspase-8 |
| **2C30** | Serine/threonine-protein kinase PAK 6 |
| **2C3Q** | Glutathione S-transferase theta-1 |
| **2C47** | Casein kinase I isoform gamma-2 |
| **2C6C** | Glutamate carboxypeptidase 2 |
| **2C6Q** | GMP reductase 2 |
| **2C9V** | Superoxide dismutase [Cu-Zn] |
| **2CAB** | Carbonic anhydrase 1 |
| **2CFY** | Thioredoxin reductase 1, cytoplasmic |
| **2CG5** | L-aminoadipate-semialdehyde dehydrogenase-phosphopantetheinyl transferase |
| **2CKG** | Sentrin-specific protease 1 |
| **2CYK** | Interleukin-4 |
| **2CZH** | Inositol monophosphatase 2 |
| **2E8A** | Heat shock protein HSP 70 |
| **2EU9** | Dual specificity protein kinase CLK3 |
| **2F2S** | Acetyl-CoA acetyltransferase, mitochondrial |
| **2F57** | Serine/threonine-protein kinase PAK 7 |
| **2F9Q** | Cytochrome P450 2D6 |
| **2FGI** | Basic fibroblast growth factor receptor 1 |
| **2FKY** | Kinesin-like protein KIF11 |
| **2FOJ** | Ubiquitin carboxyl-terminal hydrolase 7 |
| **2G1N** | Renin |
| **2GK1** | Beta-hexosaminidase subunit alpha |
| **2GLQ** | Alkaline phosphatase, placental type |
| **2GU8** | cAMP-dependent protein kinase catalytic subunit alpha |
| **2H11** | Thiopurine S-methyltransferase |
| **2HGS_1** | Glutathione synthetase |
| **2HGS_2** | Glutathione synthetase |
| **2HHA** | Dipeptidyl peptidase 4 |
| **2HI4** | Cytochrome P450 1A2 |
| **2HRB** | Carbonyl reductase [NADPH] 3 |
| **2IIP** | Nicotinamide N-methyltransferase |
| **2ILK** | Interleukin-10 |
| **2J0D** | Cytochrome P450 3A4 |
| **2J4E** | Inosine triphosphate pyrophosphatase |
| **2NNQ** | Fatty acid-binding protein, adipocyte |
| **2NZ2** | Argininosuccinate synthase |
| **2O05** | Spermidine synthase |
| **2O23** | 3-hydroxyacyl-CoA dehydrogenase type-2 |
| **2OBD** | Cholesteryl ester transfer protein |
| **2OJ9** | Insulin-like growth factor 1 receptor |
| **2ORV** | Thymidine kinase, cytosolic |
| **2PFR** | Arylamine N-acetyltransferase 2 |
| **2PK4** | Plasminogen |
| **2QY0** | Complement C1r subcomponent |
| **2QYK** | cAMP-specific 3',5'-cyclic phosphodiesterase 4A |
| **2VQM** | Histone deacetylase 4 |
| **2Z5F** | Sulfotransferase family cytosolic 1B member 1 |
| **2Z5X** | Amine oxidase [flavin-containing] A |
| **2Z7R** | Ribosomal protein S6 kinase alpha-1 |
| **3BWY** | Catechol O-methyltransferase |
| **3C0Z** | Histone deacetylase 7 |
| **3CQW** | RAC-alpha serine/threonine-protein kinase |
| **3DYD** | Tyrosine aminotransferase |
| **3JDW** | Glycine amidinotransferase, mitochondrial |
| **4GTU** | Glutathione S-transferase Mu 4 |
| **5GAL** | Galectin-7 |
| **5P21** | GTPase HRas |

Numbers following each PDB ID denote the pocket number.
