# Supplementary material for: Identifying Unexpected Therapeutic Targets via Chemical-Protein Interactome
Source: PLoS One. 2010 Mar 8;5(3):e9568. doi: 10.1371/journal.pone.0009568 (PMC2833192; doi:10.1371/journal.pone.0009568)
Supplement: Table S2 — Interactome of probe molecules towards the tree proteins. (0.13 MB DOC) [file pone.0009568.s002.doc]

**Table S2.** Interactome of probe molecules towards the tree proteins.

| **Drug name** | **AChE** | **HDAC7** | **HLA-B*5703** | **Molecule type** |
| --- | --- | --- | --- | --- |
| Acetylcholine | -4.40 | -4.79 | -3.39 | Substrate of AChE |
| Donepezil | -8.14 | -8.20 | -8.05 | AD drug molecule |
| Galantamine | -8.24 | -8.00 | -7.48 | AD drug molecule |
| Galantamine 2 | -8.24 | -7.93 | -6.94 | AD drug molecule |
| Huperzine_a | -8.57 | -7.73 | -5.04 | AD drug molecule |
| Memantine | -6.92 | -6.93 | -4.77 | AD drug molecule |
| Memantine2 | -6.71 | -5.81 | -4.89 | AD drug molecule |
| Physostigmine | -7.88 | -7.67 | -7.43 | AD drug molecule |
| Physostigmine2 | -7.82 | -7.64 | -6.31 | AD drug molecule |
| Rivastigmine | -7.11 | -6.52 | -7.00 | AD drug molecule |
| Tacrine | -6.83 | -6.16 | -7.92 | AD drug molecule |
| Trichostatin A | -8.26 | -7.02 | -7.10 | Inhibitor of HDAC |
| SAHA | -8.11 | -6.91 | -6.23 | Inhibitor of HDAC |
| 4_hydroxydiphenylhydantoin | -7.93 | -6.35 | -5.50 | control probe |
| 5_aminosalicylic_acid | -4.32 | -3.48 | -5.11 | control probe |
| 5_hydroxyrofecoxib | -8.35 | -7.05 | -7.68 | control probe |
| Allopurinol_new | -5.02 | -4.26 | -4.22 | control probe |
| Amoxicillin1 | -8.82 | -5.43 | -5.21 | control probe |
| Amoxicillin2 | -8.17 | -5.37 | -5.84 | control probe |
| Amphetamine | -6.13 | -7.57 | -5.54 | control probe |
| Amphetamine2 | -6.21 | -7.29 | -5.40 | control probe |
| Atorvastatin2 | -6.47 | -2.92 | -6.27 | control probe |
| Celecoxib | -7.89 | -6.04 | -6.20 | control probe |
| Cephalexin | -7.87 | -6.77 | -7.76 | control probe |
| Diclofenac | -6.61 | -5.54 | -6.18 | control probe |
| Diclofenac2 | -6.98 | -4.50 | -4.95 | control probe |
| Didanosine | -6.79 | -5.09 | -6.87 | control probe |
| Didanosine2 | -6.25 | -0.40 | -2.96 | control probe |
| Doxycycline | -7.85 | -8.03 | -6.08 | control probe |
| Doxycycline2 | -4.54 | -7.87 | -6.14 | control probe |
| Ethambutol | -5.05 | -4.95 | -3.26 | control probe |
| Ethambutol2 | -5.30 | -5.13 | -2.33 | control probe |
| Ethosuximide | -4.74 | -4.93 | -4.54 | control probe |
| Ethosuximide2 | -4.94 | -4.88 | -4.78 | control probe |
| Fluconazole | -6.19 | -5.04 | -4.65 | control probe |
| Flunitrazepam | -7.33 | -6.03 | -6.75 | control probe |
| Fluoxetine | -8.27 | -6.98 | -5.14 | control probe |
| Fluoxetine2 | -8.59 | -9.48 | -6.18 | control probe |
| Fluvastatin | -8.65 | -4.35 | -6.54 | control probe |
| Ibuprofen2 | -5.36 | -4.52 | -7.64 | control probe |
| Isoflurane | -2.74 | -3.28 | -2.44 | control probe |
| Lovastatin | -9.50 | -7.20 | -8.34 | control probe |
| Lovastatin2 | -9.14 | -6.67 | -8.78 | control probe |
| Luminal_sodium | -6.70 | -5.14 | -4.64 | control probe |
| Minocycline | -5.42 | -7.80 | -6.06 | control probe |
| Minocycline2 | -6.00 | -7.79 | -6.83 | control probe |
| Nevirapine | -7.67 | -7.56 | -5.69 | control probe |
| Piroxicam | -9.20 | -7.08 | -8.00 | control probe |
| Piroxicam2 | -8.29 | -6.33 | -7.11 | control probe |
| Risperidone | -11.69 | -9.83 | -9.09 | control probe |
| Risperidone2 | -11.39 | -8.29 | -9.04 | control probe |
| Rofecoxib | -8.70 | -8.32 | -7.96 | control probe |
| Succinylcholine | -5.81 | -5.50 | -3.39 | control probe |
| Succinylcholine2 | -3.36 | -3.16 | -3.39 | control probe |
| Sulfadoxine | -6.58 | -4.99 | -6.18 | control probe |
| Sulfamethoxazole | -6.87 | -6.85 | -6.67 | control probe |
| Sulfapyridine | -6.93 | -6.61 | -7.06 | control probe |
| Sulfasalazine | -7.93 | -6.94 | -6.50 | control probe |
| Sulfasalazine2 | -8.42 | -7.17 | -6.84 | control probe |
| Tenoxicam | -6.56 | -5.87 | -5.88 | control probe |
| Tenoxicam2 | -7.37 | -7.44 | -6.04 | control probe |
| Valdecoxib | -8.11 | -6.88 | -8.14 | control probe |
| Valproate | -4.13 | -3.16 | -5.14 | control probe |

The PDB IDs of the representative models of three proteins are 1F8U, 3C0Z and 2BVP respectively. Drug names followed by the numbers represent the derivative of this drug. Assumed normality and equal variances of docking scores among three groups could not be denied in statistical tests.
