# Supplementary material for: Identifying Unexpected Therapeutic Targets via Chemical-Protein Interactome
Source: PLoS One. 2010 Mar 8;5(3):e9568. doi: 10.1371/journal.pone.0009568 (PMC2833192; doi:10.1371/journal.pone.0009568)
Supplement: Table S3 — Interactome and correlations of docking scores among AChE and other members of HDAC family towards 13 probes. (0.04 MB DOC) [file pone.0009568.s003.doc]

**Table S3.** Interactome and correlations of docking scores among AChE and other members of HDAC family towards 13 probes.

|  | **AChE** | **HDAC8** | **HDAC4** | **HST2** |
| --- | --- | --- | --- | --- |
| Acetylcholine | -4.40 | -4.02 | -3.73 | -4.02 |
| Donepezil | -8.14 | -7.84 | -7.26 | -8.70 |
| Galantamine | -8.24 | -7.13 | -6.33 | -7.80 |
| Galantamine | -8.24 | -8.10 | -6.47 | -7.86 |
| huperzine_A | -8.57 | -6.70 | -5.89 | -7.08 |
| Memantine | -6.92 | -5.64 | -6.32 | -7.30 |
| Memantine2 | -6.71 | -5.21 | -5.51 | -6.69 |
| Physostigmine | -7.88 | -6.86 | -6.01 | -8.71 |
| Physostigmine2 | -7.82 | -6.84 | -6.24 | -8.40 |
| rivastigmine | -7.11 | -5.87 | -5.54 | -7.03 |
| Tacrine | -6.83 | -5.84 | -5.82 | -6.87 |
| Trichostatin A | -8.26 | -7.21 | -5.37 | -7.77 |
| SAHA | -8.11 | -5.66 | -5.74 | -6.70 |
| *PCC with AChE* | 1 | 0.84 | 0.74 | 0.82 |
| *p-value* |  | 2.8E-4^**^ | 0.006^**^ | 0.001^**^ |

PDB ID of the representative models of AChE, HDAC8, HDAC4 and HST2 are 1F8U, 1VKG, 2VQV and 1Q1A.

^**^Correlation is significant at the 0.01 level (2-tailed), and the null hypothesis is that the PCC equals zero.
