# Supplementary material for: Identifying Unexpected Therapeutic Targets via Chemical-Protein Interactome
Source: PLoS One. 2010 Mar 8;5(3):e9568. doi: 10.1371/journal.pone.0009568 (PMC2833192; doi:10.1371/journal.pone.0009568)
Supplement: Table S4 — Sixty two schizophrenia drug molecules. (0.05 MB DOC) [file pone.0009568.s004.doc]

**Table S4.** Sixty two schizophrenia drug molecules

| Olanzapine |
| --- |
| Olanzapine_2 |
| Clozapine |
| Clozapine_2 |
| Chlorpromazine |
| Haloperidol |
| Thioridazine |
| Risperidone |
| Risperidone_2 |
| Risperidone_3 |
| Risperidone_4 |
| Quetiapine |
| Chlorprothixene |
| Ziprasidone |
| Ziprasidone_2 |
| Sulpiride |
| Sulpiride_2 |
| Loxapine |
| Loxapine_2 |
| Remoxipride |
| Remoxipride_2 |
| Remoxipride_3 |
| Remoxipride_4 |
| Propiomazine |
| Propiomazine_2 |
| Flupenthixol |
| Flupenthixol_2 |
| Flupenthixol_3 |
| Flupenthixol_4 |
| Flupenthixol_5 |
| Flupenthixol_6 |
| Flupenthixol_7 |
| Flupenthixol_8 |
| Mesoridazine |
| Mesoridazine_2 |
| Aripiprazole |
| Aripiprazole_2 |
| Paliperidone |
| Paliperidone_2 |
| Paliperidone_3 |
| Paliperidone_4 |
| Methotrimeprazine |
| Methotrimeprazine_2 |
| Molindone |
| Molindone_2 |
| Thioproperazine |
| Thiothixene |
| Thiothixene_2 |
| Zuclopenthixol |
| Zuclopenthixol_2 |
| Zuclopenthixol_3 |
| Zuclopenthixol_4 |
| Zuclopenthixol_5 |
| Zuclopenthixol_6 |
| Zuclopenthixol_7 |
| Zuclopenthixol_8 |
| Fluspirilene |
| Fluspirilene_2 |
| Sertindole |
| Sertindole_2 |
| Sertindole_3 |
| Sertindole_4 |

Drug names followed by the numbers represent the derivative of this drug.
