# Supplementary figures and images for: Identifying Unexpected Therapeutic Targets via Chemical-Protein Interactome
Source: PLoS One. 2010 Mar 8;5(3):e9568. doi: 10.1371/journal.pone.0009568 (PMC2833192; doi:10.1371/journal.pone.0009568)

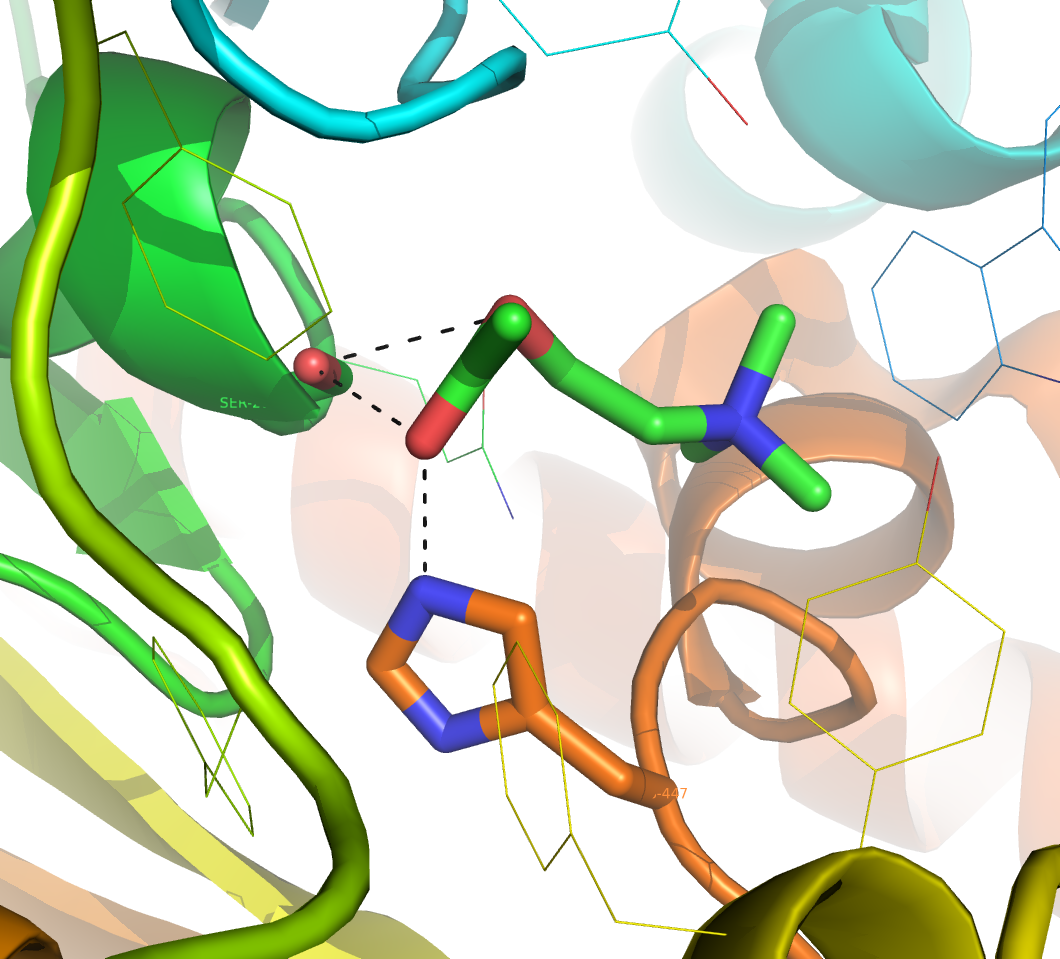

Supplement: Figure S1 — Visualization of the docking result of acetylcholine's interacting with the active center of AChE using AutoDock. The acetyl of acetylcholine interacts with two catalyzing residues (Ser203 and His447), which is accommodate to the catalytic mechanism of AChE. (1.52 MB TIF) [file pone.0009568.s005.tif]

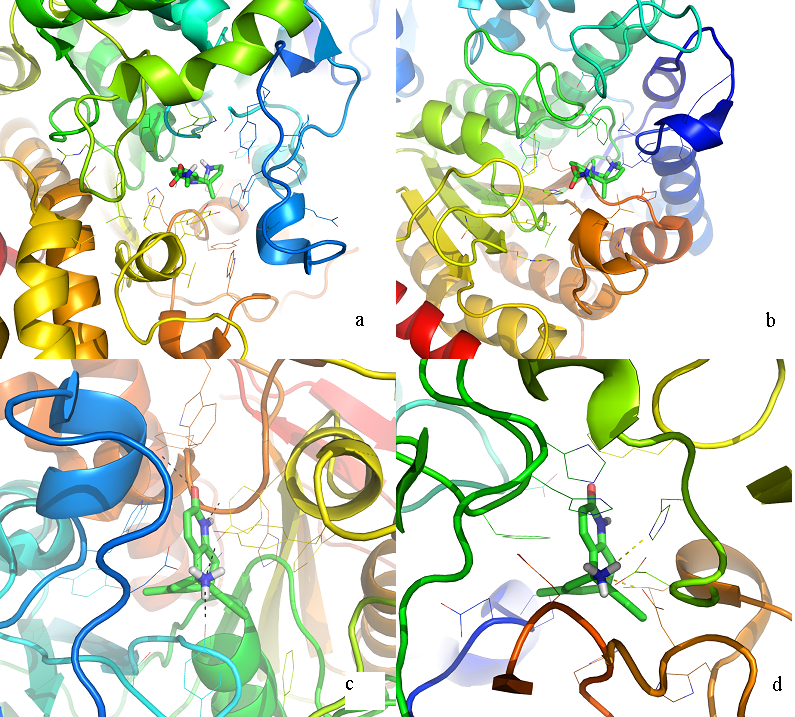

Supplement: Figure S2 — Comparison of the binding site and sequence identity in active site of AChE and HDAC7. (a, b) Comparison of the docking result of physostigmine to the active site of human AChE and hunan HDAC7. (c, d) Comparison of the docking result of huperzine A to the active site of human AChE and hunan HDAC7. Residues within 6Å of the docked ligand of AChE (PDB ID: 1F8U) are Asp74, Gly82, Thr83, Met85, Trp86, Gly120, Gly121, Gly122, Phe123, Tyr124, Ser125, Gly126, Leu130, Tyr133, Gln202, Ser203, Ala204, Phe295, Phe297, Tyr337, Phe338, Tyr341, Trp439, Pro446, His447, Gly448 and Tyr449. Residues within 6Å of the docked ligand of HDAC7 (PDB ID: 3Z0Y) are His541, Pro542, Glu543, His544, Arg547, Asp626, Pro667, His669, His670, Gly678, Phe679, Asp707, Val708, His709, Phe738, Gly799, Phe800, Asp801, His806, Pro809, Leu810, Gly811, Glu840, Gly841, Gly842 and His843. No significant similarity could be observed within these amino acids between the two proteins. (0.87 MB TIF) [file pone.0009568.s006.tif]
