# Supplementary material for: Identifying Unexpected Therapeutic Targets via Chemical-Protein Interactome
Source: PLoS One. 2010 Mar 8;5(3):e9568. doi: 10.1371/journal.pone.0009568 (PMC2833192; doi:10.1371/journal.pone.0009568)
Supplement: File S1 — Comparing the variances introduced by the ligands and proteins respectively. Deducing procedures of elimination of the protein and the ligand factors. (0.08 MB DOC) [file pone.0009568.s007.doc]

**File S1**

To give a reasonable explanation to why Z’-score, not docking score, is suitable to represent chemical-protein interactions, we put forward a linear model that a docking score X_ij_ can be factorized as:

$X_{ij}=\mu+\alpha_{i}+\beta_{j}+(\alpha\beta)_{ij}$ , (1)

where μ is the mean of docking scores, α_i_ and β_j_ are the assessment of the endogenous contributions of protein factor i and ligand factor j respectively; (αβ)_ij_ is the interactive effect of the two factors, which reflects the nature of the chemical-protein affinity. Both the ligand and the protein factors are considered as the random effect. Comparing the variances of these effects within a test CPI, we found that the protein and the ligand effects contributed largely to the variances of the docking scores, and dominated the interactive effect significantly.

The type IV method was used to calculate the sum of squares. The normality of the chemical-protein interactive effect was guaranteed by the Kolmogorov-Smirnov test. The F value was computed as MS_c_ / MS_cp_ and MS_p_ / MS_cp_ respectively, where MS_c_, MS_p_ and MS_cp_ denoted the mean squares of the ligand, protein and the interactive effects.

The mean squares ratios (F values) of the protein and the ligand effect towards the interactive effect are 11.1 (p = 4.2E-67) and 65.8 (p = 0) respectively (see the table below).

Tests of between-subjects effects of the testing CPI.

| Source | | Type IV Sum of Squares | df | Mean Square | F | Sig. |
| --- | --- | --- | --- | --- | --- | --- |
| Protein |  | 38108.290 | 45^a^ | 846.851 | 11.092 | 4.2E-67 |
|  |  |  |  |  |  |  |
| Ligand |  | 215924.105 | 43^a^ | 5021.491 | 65.771 | 0.000 |
|  |  |  |  |  |  |  |
| Protein * Ligand |  | 133532.162 | 1749 | 76.348 | . | . |
|  | Error | .000 | 0 ^b^ | . |  |  |

^a^ Some of the proteins and were excluded from this test due to their high missing value rate.

^b^ The errors could not be estimated since the docking score of a ligand-protein pair would remain unchanged whatever times the docking procedures were performed.

After applying 2DIZ transformation however, the protein and the ligand factors are eliminated and the Z’-score is determined by the interactive effects (see deducing procedures below):

let $W_{ij}=X_{ij}-\overline{X_{i}}=\alpha_{i}-\frac{1}{m}\sum_{k=1}^{m} \alpha_{k}+(\alpha\beta)_{ij}-\frac{1}{m}\sum_{k=1}^{m} (\alpha\beta)_{kj}$,

thus $Z_{ij}-\overline{Z_{i}}=(\frac{W_{ij}}{SD_{X_{j}}}-\frac{1}{n}\sum_{k=1}^{n} \frac{W_{ik}}{SD_{X_{k}}})$,

where n and m are the number of ligands and proteins respectively. In this linear model, the equal of variance is assumed, given that the constant $a=SD_{X_{1}}=SD_{X_{2}}=...=SD_{X_{k}}$, then:

$\begin{matrix} \\ Z_{ij}-\overline{Z_{i}}=\frac{1}{a}\left\{\alpha_{i}-\frac{1}{m}\sum_{k=1}^{m} \alpha_{k}+(\alpha\beta)_{ij}-\frac{1}{m}\sum_{k=1}^{m} (\alpha\beta)_{kj}-\frac{1}{n}\left[n\alpha_{i}-\frac{n}{m}\sum_{k=1}^{m} \alpha_{k}+\sum_{k=1}^{n} (\alpha\beta)_{ik}-\frac{1}{m}\sum_{q=1}^{n} \sum_{k=1}^{m} (\alpha\beta)_{kq}\right]\right\} \\ =\frac{1}{a}\left[(\alpha\beta)_{ij}-\frac{\sum_{k=1}^{m} (\alpha\beta)_{kj}}{m}-\frac{\sum_{k=1}^{n} (\alpha\beta)_{ik}}{n}-\frac{\sum_{q=1}^{n} \sum_{k=1}^{m} (\alpha\beta)_{kq}}{mn}\right] \\ \end{matrix}$since the item $\frac{\sum_{q=1}^{n} \sum_{k=1}^{m} (\alpha\beta)_{kq}}{mn}$ is a constant, let $b=\frac{\sum_{q=1}^{n} \sum_{k=1}^{m} (\alpha\beta)_{kq}}{mn}$, thus

$Z_{ij}-\overline{Z_{i}}=\frac{1}{a}\left[(\alpha\beta)_{ij}-\frac{\sum_{k=1}^{m} (\alpha\beta)_{kj}}{m}-\frac{\sum_{k=1}^{n} (\alpha\beta)_{ik}}{n}-b\right]$.

According to our model, ligand j only interacts with protein i, so $(\alpha\beta)_{ij}=0$ when i ≠ j. Consequently,

$Z_{ij}-\overline{Z_{i}}=-\frac{1}{a}\left[\frac{(\alpha\beta)_{jj}}{m}+\frac{(\alpha\beta)_{ii}}{n}+b\right],i\neq j$;

$Z_{ij}-\overline{Z_{i}}=\frac{1}{a}\left[(\alpha\beta)_{ij}-\frac{(\alpha\beta)_{ij}}{m}-\frac{(\alpha\beta)_{ij}}{n}-b\right],i=j$.

So the standard deviation of the Z’-scores for protein i is:

, and finally, the Z’-score is:

$$Z'_{ij}=\frac{Z_{ij}-\overline{Z_{i}}}{SD_{Z_{i}}}=\frac{-\sqrt{n-1}\left[\frac{(\alpha\beta)_{jj}}{m}+\frac{(\alpha\beta)_{ii}}{n}+b\right]}{\sqrt{(n-1)\left[\frac{(\alpha\beta)_{jj}}{m}+\frac{(\alpha\beta)_{ii}}{n}+b\right]^{2}+\left[(1-\frac{1}{m}-\frac{1}{n})(\alpha\beta)_{ii}-b\right]^{2}}},i\neq j$$

$\begin{matrix} Z'_{ij}=\frac{Z_{ij}-\overline{Z_{i}}}{SD_{Z_{i}}}=\frac{\sqrt{n-1}\left[(\alpha\beta)_{ij}(1-\frac{1}{m}-\frac{1}{n})-b\right]}{\sqrt{(n-1)\left[\frac{(\alpha\beta)_{jj}}{m}+\frac{(\alpha\beta)_{ii}}{n}+b\right]^{2}+\left[(1-\frac{1}{m}-\frac{1}{n})(\alpha\beta)_{ij}-b\right]^{2}}},i=j \\ \end{matrix}$.

Consequently,

$Z'_{ij}=\frac{-b\sqrt{n-1}}{\sqrt{(n-1)b^{2}+\left[(\alpha\beta)_{ii}-b\right]^{2}}}$ $(i\neq j)$, when $(m\to+\infty,n\to+\infty)$

$Z'_{ij}=\left[(\alpha\beta)_{ij}-b\right]\sqrt{\frac{(n-1)}{(n-1)b^{2}+\left[(\alpha\beta)_{ij}-b\right]^{2}}}$ $(i=j)$, when $(m\to+\infty,n\to+\infty)$.
